# Treatment Resistant Patients with Metabolic Dysfunction-associated Steatohepatitis: Long-term Follow-up Prospective Study

**DOI:** 10.31662/jmaj.2024-0371

**Published:** 2025-04-04

**Authors:** Masayuki Tsujisaki, Takenori Takamura, Hideyasu Takagi, Seiya Nakahara, Mamiko Suwa, Hideto Itoh, Noriyuki Akutsu, Shigeru Sasaki, Hiroshi Nakase

**Affiliations:** 1 Department of Gastroenterology, Tenshi Hospital, Sapporo, Japan; 2 Department of Gastroenterology and Hepatology, Sapporo Medical University, Sapporo, Japan

**Keywords:** metabolic dysfunction-associated steatohepatitis (MASH), treatment resistant group, long-term follow-up study, pemafibrate, sodium-glucose cotransporter2 (SGLT2) inhibitor, vitamin E, multidrug therapy

## Abstract

**Introduction:**

Many treatments for patients with metabolic dysfunction-associated steatohepatitis (MASH) have been proposed; however, most studies showed the results for a single medication and a short duration of treatment. The long-term outcomes of the multidrug therapies remain indeterminate. We conducted a study to investigate the usefulness of multidrug combination therapy for every kind of MASH patient and the differences between treatment-sensitive and treatment-resistant patients.

**Methods:**

Fifty-one patients (middle-aged, in their 40s to 60s, metabolic generation) with MASH-determined fibrosis staging were enrolled. Primary treatment (weight control and medication of vitamin E and sodium-glucose cotransporter 2 inhibitor (SGLT2i)) was done and then pemafibrate treatment was added.

**Results:**

Regarding responses to the step-by-step multidrug therapy, patients with MASH were divided into 3 groups, with use of 3 markers―alanine aminotransferase (ALT) (hepatitis), elasticity value (E value, liver stiffness measurement) (hepatitis/fibrosis), and type IV collagen (fibrosis); group 1: sensitive to primary treatment (n = 35), group 2: resistant to primary treatment and sensitive to pemafibrate treatment (n = 11), and group 3: resistant to both treatments (n = 5).

To determine the parameters related to treatment resistance, the baseline levels of parameters_―_obesity (body mass index), metabolic factor (visceral fat, controlled attenuation parameter), diabetes mellitus (DM) (glycated hemoglobulin (HbA1_c_), fasting immunoreactive insulin), lipid metabolism (triglyceride), and hepatitis (ALT)―were compared between treatment-sensitive group 1+group 2 and treatment-resistant group 3. However, none of them had differences statistically. The same analysis showed that type IV collagen, E value, FIB-4 index (age (year) x AST (IU/L)/platelet count (10^4^/L) x ALT (IU/L)^1/2^), and MASH fibrosis had differences statistically.

**Conclusions:**

The most effective treatment for patients with MASH could not be determined, according to the baseline levels of characteristics; however, weight control and step-by-step multidrug therapies made it possible to stabilize more than 90% of patient conditions and to solve MASH without worsening fibrosis. Since high levels of liver fibrosis-related markers affected the treatment resistance, MASH treatments should be started in an early stage while the levels of each marker are still low; type IV collagen <5.3 ng/mL, E value <13.7 kPa, FIB-4 index <1.89 and MASH fibrosis stage 2 or less.

## Introduction

Metabolic dysfunction-associated steatotic liver disease (MASLD) has attracted worldwide attention due to the increasing number of patients ^(1), (2)^. Metabolic dysfunction-associated steatohepatitis (MASH; 10%~20% of MASLD) is a progressive liver disease from steatohepatitis to cirrhosis and hepatocellular carcinoma ^(3), (4)^. Although bodyweight reduction by lifestyle measures including a food diary and exercise is beneficial for MASH patients, it is difficult to achieve and maintain over time. Therefore, effective therapeutic options for patients with MASH to suppress the progression of the disease are needed. Many drugs with different mechanisms of action have been studied and evaluated. Among them, vitamin E ^(5), (6)^, pioglitazone ^(5), (7)^, sodium-glucose cotransporter 2 (SGLT2) inhibitors ^(8), (9)^, and pemafibrate ^(10), (11), (12)^ have been shown to be safe, effective and available to use. Currently, there is no approved pharmacotherapy for MASH, and no efficacy data exist on long-term survival and clinical outcomes besides the result of vitamin E ^(6)^. Most studies on MASH treatment have only reported the results of the improvement in liver histology and surrogate markers for short (1~2 years)-term and with a single medicine, compared to control. In a longitudinal study of patients, fibrosis stage, but no other histologic features of steatohepatitis, was independently associated with long-term overall mortality and liver-related events ^(13)^. From the above, it is most important to confirm whether the treatments for all kinds of patients with MASH can make it possible to stabilize liver inflammation and suppress fibrosis, utilizing a combination of drugs with different mechanisms of action.

We conducted a study to investigate: (1) the usefulness of multidrug treatment, (2) the effective rate for maintaining a stable state against fibrosis progression, (3) the differences between the treatment-sensitive group and the treatment-resistant group.

## Material and Methods

### Study population and data collection

This is a retrospective observational study that enrolled patients with MASLD/MASH between May 2015 and December 2021.

Of the patients who had liver dysfunction or had been diagnosed with fatty liver, chronic hepatitis, and liver cirrhosis, 132 patients who had liver biopsy-confirmed MASLD or were diagnosed with positive pattern recognition system ^(14)^ were included. MASLD/MASH was diagnosed ^(1), (2)^ based on the presence of fatty liver. Daily alcohol intakes were <30 g/day and <20 g/day in male and female patients, respectively. None of the patients received any medications that cause steatotic liver disease. All patients met at least one of the following cardio-metabolic criteria in the MASLD definition ^(1), (15)^: (1) body mass index (BMI) ≥23 kg/m^2^, waist circumference ≥94 cm (male), ≥80 cm (female); (2) fasting serum glucose ≥100 mg/dL, or 2-h post-lord glucose ≥140 mg/dL, or glycated hemoglobin (HbA1c) ≥5.7, or type 2 diabetes, or ongoing treatment for type 2 diabetes; (3) blood pressure ≥130/85 or ongoing specific antihypertensive drug treatment; (4) plasma triglyceride ≥150 mg/dL, or ongoing lipid-lowering treatment; and (5) plasma high density lipoprotein (HDL)-cholesterol ≤40 mg/dL (male), ≤50 mg/dL (female), or ongoing low HDL-cholesterol treatment. According to a new classification of steatotic liver disease, patients with the following diseases were excluded: alcohol-related liver disease, drug-induced liver disease, autoimmune hepatitis, viral hepatitis, secondary steatotic liver disease, primary biliary cholangitis, and Wilson’s disease.

Of 132 MASH patients, 51 middle-aged (40-60 years old) MASH patients were enrolled in this study, because these patients are thought to be metabolic generations and to be suitable for the analysis of treatment efficacy.

For body measurements, BMI and visceral fat (VF) measured by computed tomography (CT) imaging were used. The controlled attenuation in parameter (CAP) ^(16)^ specifically targets hepatic steatosis using a process based on Fibroscan (Echoscan, France). As biochemical data, HbA1c, fasting immunoreactive insulin (f-IRI; diabetes mellitus [DM]), triglyceride, low density lipoprotein (LDL) cholesterol (lipid metabolism), alanine aminotransferase (ALT) (hepatitis), and type IV collagen (liver fibrosis) were selected. Liver stiffness measurement (elasticity value [E value], kPa ^(17)^) was measured using Fibroscan as a liver elastic marker for liver fibrosis/hepatitis. MASH stages, since FIB-4 index (age (year) x AST (IU/L)/platelet count (10^4^/L) x ALT (IU/L)^1/2^ ) was selected to analyze the MASH characterization associated with treatment resistance (Table 1, group 1 + 2 + 3).

**Table 1.** Clinical Characteristics of Patients with MASH.

Characteristics	Group1+2+3	Group1	Group2	Group3
	(n=51）	(n=35)	(n=11)	(n=5)
Age --- yrs	49.8±6.3	50.1±6.5	47.3±5.1	53.4±5.0
Male --- no. (%)	27 (53)	20 (57)	6 (55)	1 (20)
Body measurement				
Body mass index	31.2±4.9	31.7±4.6	30.9±5.7	29.1±4.3
Visceral fat cm2	199.0±63.1	202.2±67.0	205.9±54.0	165.6±41.9
Diabetes Mellitus (DM%)	(51%)	(49%)	(55%)	(60%)
HbA1c	6.7±1.3	6.7±1.4	6.7±1.1	6.4±0.5
f-IRI	18.8±7.7	17.9±6.3	21.7±10.4	21.2±3.4
Lipid metabolism				
Triglyceride (hyperTG %)	242±257 (55%)	223±290 (45%)	292±178 (64%)	260±98 (100%)
LDL cholesterol (hyperLDL％)	131±27 (39%)	127±28 (34%)	136±11 (55%)	149±33 (40%)
Biochemical data				
ALT (Hepatitis)	97.5±52.5	97.5±58.4	99.2±29.7	93.4±51.6
Diagnostic imaging (FibroScan)				
E value (fibrosis/hepatitis)	10.2±6.2	9.5±4.9	8.7±4.9	17.8±10.0
CAP (Steatosis)	321.4±44.0	321.7±45.4	325.9±32.3	310±54.0
Liver fibrosis				
Type IV collagen 4s	4.9±1.7	4.6±1.3	5.1±1.8	7.3±1.8
MASH stage (1/2/3/4) no.	33/9/9/0	24/7/4/0	8/1/2/0	0/2/3/0
FIB4-index	1.67±0.89	1.51±0.64	1.33±0.56	3.16±1.09

Results are expressed as means ±SD or as members with percentages. F IRI fasting immunireactive insulin, ALT alanine aminotransferase, E value elasticity value, CAP controlled attenuation parameter Group1: primary treatment sensitive group (n=35) Group2: primary treatment resistant and pemafibrate treatment sensitive group (n=11) Group3: both treatments resistant group (n=5)

### Primary and final endpoint

The primary endpoints of this study were the changes in measured variables from baseline. Since hepatic inflammation plays a central role in the progression to advanced fibrosis ^(18)^, it is important to suppress inflammation with the use of many means and multidrug therapy. Moreover, liver fibrosis is associated with liver-related mortality and overall survival ^(13)^. The primary endpoint is to increase the effectiveness of treatment and to investigate its causes.

**Figure 1.**
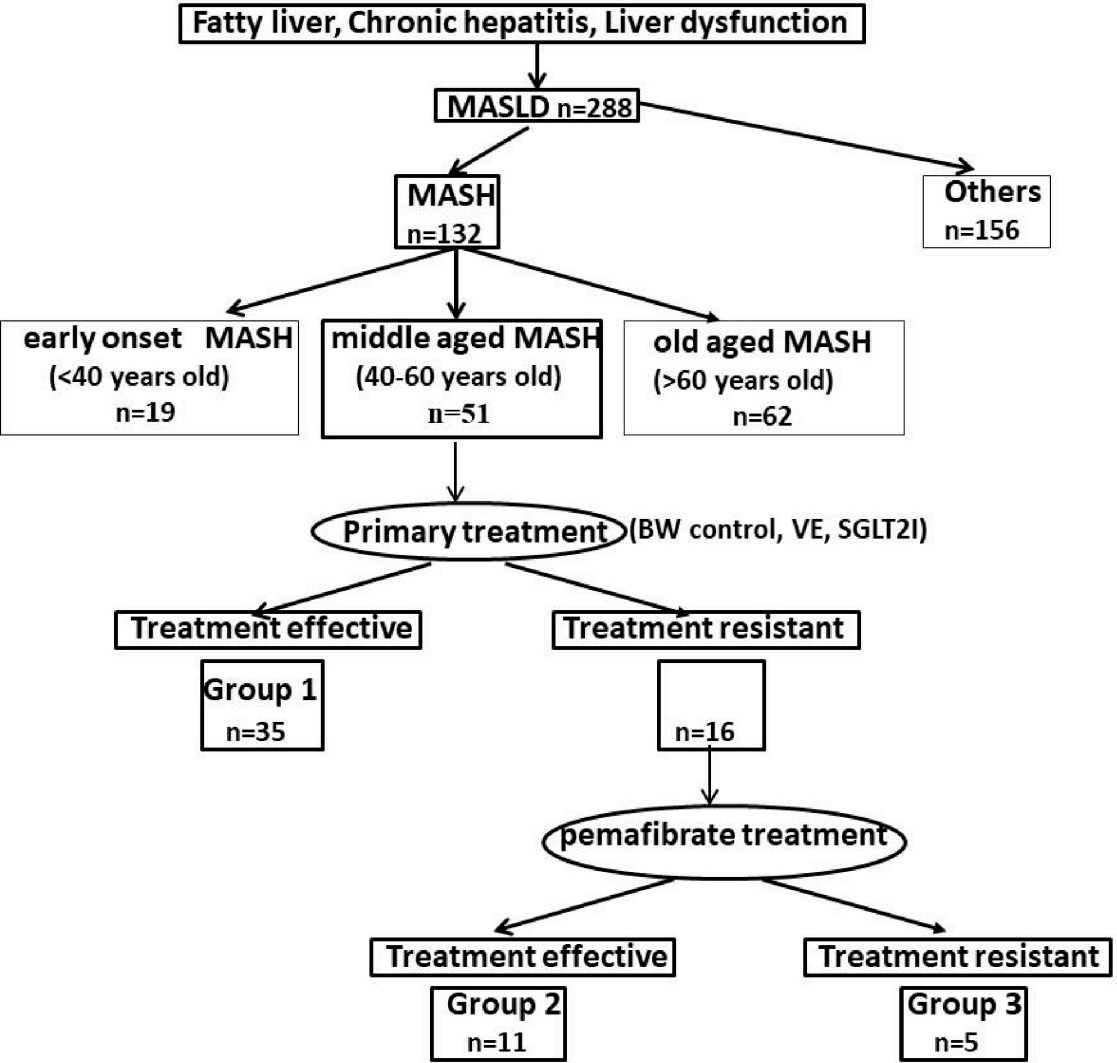
A schematic representation of the study design.

**Figure 2.**
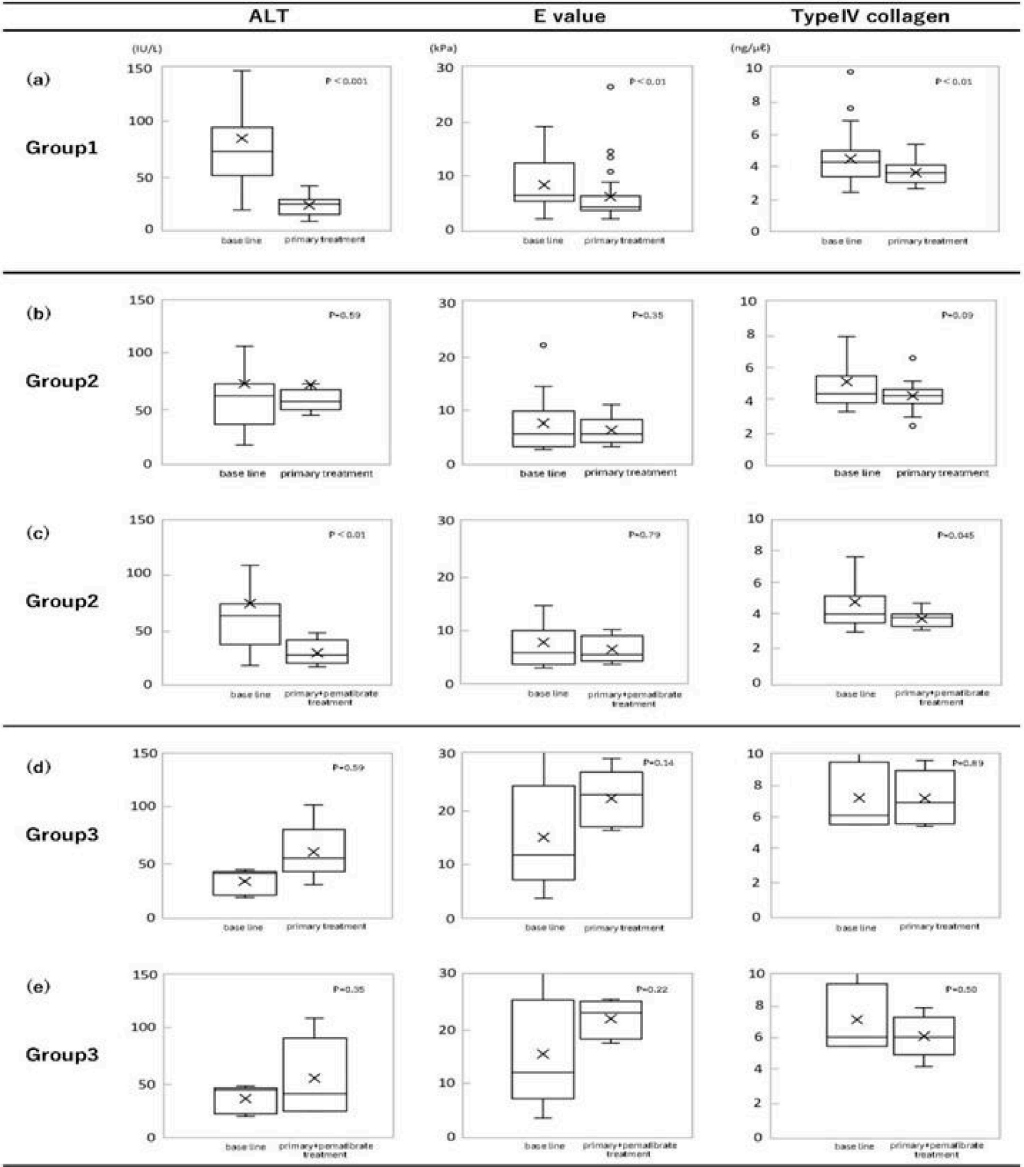
Therapeutic effects of primary treatment and pemafibrate treatment evaluating by using 3 markers (ALT, E value, and type IV collagen), Boxplots (median, upper and lower quartiles, range and Outliers [circles]) of ALT, E value, and type IV collagen in group 1a, group 2b, 2c and group 3d, 3e. (a) A statistical improvement of all markers was observed in sensitive group 1 after primary treatment. Note: p-values were calculated by Wilcoxon’s signed-rank test (p < 0.001 of ALT, p < 0.01 of E value, and p < 0.01 of type IV collagen) and primary treatment was statistically effective. (b) No improvement was observed in group 2 after primary treatment. Note: p-values were calculated but there were not significant differences. (c) A statistical improvement was observed in group 2 after pemafibrate treatment. Note: P-values were p < 0.01 (ALT), p < 0.79 (E value) and p,0.045 (type IV collagen). (d), (e) No improvement of 3 markers was observed in treatment resistant Group 3 after primary treatment and pemafibrate one. Note: p-values were calculated, but there were not significant differences after both primary and pemafibrate treatments. ALT: alanine aminotransferase; E value: elasticity value.

Body weight (BW) control, Vitamin E, and SGLT-2 inhibitors were used as primary therapies. Primary treatment was given to middle-aged MASH patients (n = 51) for several years (Figure 1 and 2a). The duration of the treatment period was used, as follows; BW control (6-18 Months), vitamin E (12M), SGLT2 inhibitor (SGLT2i) (12M), and pemafibrate (12M) (shown in Table 2).

**Table 2.** Effectiveness Rate and Duration Time in Primary and Pemafibrate Treatment.

				Effectiveness rate (%)			Duration time (years)
	Primary treatment		pemafibrate treatment	Group 1	Group 2	Group 3	
				n=35	n=11	n=5	
1) BW				29%			2.4ys
2) BW	Vitamin E			43%			3.1ys
3) BW	(Vitamin E)	SGLT2i		17%			3.3ys
	Low effect						
4) (BW)	Vitamin E	SGLT2i		11%			5.0ys
Low effect							
5) (BW)	Vitamin E	(SGLT2i)	+pemafibrate		36%		4.4ys
Low effect		Low effect					
6) (BW)	Vitamin E	SGLT2i	+pemafibrate		64%		4.1ys
Low effect							
7) (BW)	(Vitamin E)	(SGLT2i)	(+pemafibrate)			0%	5.7ys
Low effect	Low effect	low effect	low effect				

Results of effectiveness rate are expressed as percentages of each group with different therapies. Parenthesis (drug name) means that the drug effect is not sufficient. BW body weight, SGLT2i sodium-glucose cotransporter 2 inhibitor

We selected ALT (hepatitis), E value (hepatitis and fibrosis), and type IV collagen (fibrosis) as markers for evaluation of therapeutic effect.

The criteria for treatment effectiveness in the primary and pemafibrate treatments were used about 3 markers, as follows:

ALT: the levels after treatment were improved from the baseline levels and were below 45 IU/L.

E value: those were improved or were not changed, because E values were sensitive and influenced by inflammation and fibrosis.

Type IV collagen: those were improved and were below 5.0 ng/mL.

Based on the criteria, group 1 was defined as the primary treatment-effective, comparing the levels of 3 markers after primary treatment and baseline levels (Figure 2a).

Group 2 was defined as patients who did not show improvement in 3 markers, comparing the levels after primary treatment and baseline levels, shown in Figure 2b. However, the results after additional pemafibrate treatment of group 2 showed the effectiveness (Figure 2c). Since the differences were significant, pemafibrate was suggested to be effective for group 2. Finally, group 3 (n = 5) was decided as treatment-resistant to both treatments (Figure 1, 2c and 2d).

### Statistical analysis

Continuous 3 markers (ALT, E value, and type IV collagen) were expressed as the median (interquartile range). Wilcoxon’s signed-rank test ^(11)^ was used to compare continuous values at baseline and those after the treatment. Data on the changes of 3 markers were analyzed to check treatment resistance. A p-value of <0.05 was considered to indicate statistical significance. Primary treatment effects in group 1 and primary treatment and pemafibrate treatment effects in group 2. Group 3 was studied to evaluate the treatment resistance (Figure 2).

Another interest of this study is to analyze and decide the parameters that are related to the treatment sensitivity or resistance.

If related parameters were known before treatment, it would be possible to decide the treatment targets and to start medication just on time of intervention. As clinical parameters, obesity, metabolic syndrome: BMI, VF, and CAP, DM and lipid metabolism: HbA1c, f-IRI and triglyceride (TG): hepatitis-ALT, liver fibrosis: E value, type IV collagen, and stages of MASH, and other: FIB-4 index were studied. The statistical differences between group 1 + group 2 (treatment-sensitive) and group 3 (treatment-resistant) were determined using the t-test for quantitative data (partially determined using the chi-square test). p-values were calculated and the differences were considered to be statistically significant at p < 0.05.

Multivariate analysis was conducted using logistic regression to independently identify variables associated with the treatment-effective group and those associated with the treatment-resistant group. We calculated the sensitivity and specificity to evaluate the accuracy of markers in determining treatment sensitivity and resistance. Using these results, we constructed receiver operating characteristic (ROC) curves by plotting sensitivity against 1-specificity at each value ^(19)^. Differences were considered statistically at p < 0.05.

## Results

### Patient characteristics

Between May 2015 and December 2021, 288 patients were screened by biochemical data, CT imaging, Fibroscan, and liver biopsy. Fifty-one middle-aged patients (40-60 years old) of 132 MASH patients were enrolled in this study. According to our MASH study so far, the characteristics of patients in early-onset MASH (<40 years) and those of old-aged onset MASH (>60 years) were quite different from middle-aged MASH onset (40-60 years). The general points are as follows:

Early-onset MASH patients

1) Short duration of illness (<10 years)

2) Diabetes prevalence is 10%

3) High rate of impaired glucose tolerance

4) High insulin resistance

5) Obese condition

Old-aged MASH patients

1) Long duration of illness (20-30 years)

2) Diabetes prevalence is 50%

3) Insulin resistance down

4) BW tends to down

5) Liver fibrosis stage 3, stage 4 (cirrhosis), and hepatocellular carcinoma (HCC) patients are included

As these groups are suggested to be different, the treatments are inevitably different. Simply speaking about the treatment, BW control is the best way to improve patients with early-onset MASH; however, the treatment of patients with old-aged MASH is very different and complicated. Because the old-aged group is made up of many different types of patients: fibrosis stage 3-4 (liver cirrhosis), HCC, low BW, severe diabetes, etc. It is important to study and analyze characteristics by age of MASH patients. This study focuses on middle-aged onset MASH to see typical treatment approaches before patients with MASH have treatment resistance with age up.

The baselines of patient characteristics are shown in Table 1. Because the mean age of patients was 49.8 years and male patients were 53%. Since the BMI was 31.2 kg/m^2^ and VF 199.0 cm^2^, DM 51%, hyper TG 55%, and hyper LDL cholesterol 39%, these patients were thought to belong to a metabolic generation.

### Primary treatment and pemafibrate treatment

Since it is difficult to control the pathogenesis of MASH only with a single factor, multiple means are necessary to get satisfactory results. BW control, vitamin E, an SGLT2i, pioglitazone, and pemafibrate are well-known as useful treatments. Pioglitazone was excluded, because of its side effects; promoting obese condition and heart failure. Because none of them has complete effectiveness, multidrug therapies are selected in general practice. There is no report to evaluate long-term treatment. In this study, a combination of multiple means was done to get more complete results. To evaluate the treatment sensitivity, the criteria of 3 markers to see the treatment effectiveness were set up and shown in Material and Methods. BW control, vitamin E, and SGLT2i were utilized as primary treatment and then pemafibrate was medicated to the primary treatment-resistant patients (Figure 1). It has taken several years (3.2 ± 1.9 years) to divide treatment-sensitive group 1 and others (Table 2). Based on the criteria, group 1 was defined as the primary treatment-effective, comparing the levels of 3 markers after primary treatment and baseline levels (Figure 2a). Three markers (ALT, E value, and type IV collagen) were used since treatment efficacy could be determined by the improvement of the markers. Group 1 (n = 35) showed the improved results of markers (Figure 2a). The reductions in these levels were observed, comparing between the baseline levels and those after treatment. The differences of the levels were significant; ALT (p < 0.001), E value (p < 0.01) and type IV collagen (p < 0.01). Therefore, group 1 (n = 35) patients were sensitive to the primary treatment. The proportion of treatment-sensitive patients is 69% of all (n = 51). That is, even if primary treatment was made full use of BW control, vitamin E, and SGLT2i and continued for more than 3 years, 31% (n = 16) of MASH patients remained treatment-resistant (Table 2). The next endpoints were the changes in these 3 markers after additional pemafibrate treatment.

Group 2 was defined as patients who did not show improvement in 3 markers, comparing the levels after primary treatment and baseline levels, shown in Figure 2b. However, the results after additional pemafibrate treatment of group 2 showed the effectiveness (Figure 2c). Since the differences were significant, pemafibrate was suggested to be effective for group 2.

As an additional treatment, pemafibrate was investigated for its efficacy in resistant patients (31%, n = 16). Eleven out of 16 patients (group 2) were sensitive to pemafibrate treatment. Significant reduction of ALT (p < 0.01) and type IV collagen values (p < 0.045) were observed, shown in Figure 2c. The difference in E value level was not significant between baseline and the level after pemafibrate treatment. In contrast, the difference between just before the pemafibrate treatment level and after the treatment level of E value was significant (p < 0.05). It was suggested that pemafibrate was useful because the levels of E value elevated more than the baseline during the primary treatment and then the levels were statistically improved by pemafibrate treatment. The results of group 2 (n = 11) did not improve in markers by the primary treatment (Figure 2b) and the results after pemafibrate treatment showed the improvement (Figure 2c). Pemafibrate was effective for group 2 patients. The results of group 3 (n = 5) did not improve in markers; both those after primary treatment and those after pemafibrate treatment (Figure 2d and 2e). Finally, group 3 (n = 5) was decided as treatment-resistant to both of them (Figure 1, 2d and 2e).

In summary, the primary treatment effectivity rate was 68.6% (35/51 cases) and then it changed up to 90.2% (46/51 cases) by adding pemafibrate. Finally, the treatment resistance rate became 9.8%.

The effectiveness rates of medical intervention were analyzed for 3-5 years. The treatment plans used a step-by-step approach to improve and stabilize without worsening fibrosis. The effectiveness rates of different therapies were as follows (shown in Table 2);

Primary treatment

Group 1; 1) BW control 29%, 2) BW control, vitamin E 43%,

3) BW control, (vitamin E low effect), SGLT2i 17%, 4) (BW control low effect), vitamin E, SGLT2i 11%

Pemafibrate treatment

Group 2; 5) (BW control low effect), vitamin E, (SGLT2i low effect) + pemafibrate 36%, 6) (BW control low effect), vitamin E, SGLT2i + pemafibrate 64%

Group 3: 7) (BW control low effect), (vitamin E low effect), (SGLT2i low effect) + (pemafibrate low effect) 0%

Therapeutic effects of primary treatment (group 1) and additional pemafibrate therapy (group 2 and group 3) were analyzed on representative items, using Wilcoxon’s signed-rank test. BW, HbA1c (glucose metabolism), and triglyceride (lipid metabolism) were selected to analyze as representative items. As shown in Table 3, the differences in BW, HbA1c, and triglyceride between baselines and those after primary treatment were significant, p < 0.001, p < 0.02, and p < 0.02 respectively. These results have indicated that BW reduction is suggested to be effective for all items. Another important result showed that triglyceride was improved after additional pemafibrate treatment in group 2. The only difference between baseline and those after pemafibrate treatment was significant. Pemafibrate had been of great benefit to group 2 patients.

**Table 3.** Therapeutic Effects of Primary and Pemafibrate Treatment on Representative Items; BW, Diabetes (HbA1c) and Lipid (TG).

	Body weight (kg)		HbA1c (%)		Triglyceride (mg/dl)	
	baseline	after primary treatment	baseline	after primary treatment	baseline	after primary treatment
Group1	83.1±16.0	77.8±16.4	6.7±1.3	6.4±1.1	182.2±115.5	148.8±66.7
	p<0.001		p<0.02		p<0.02	
	**baseline**	**after pemafibrate treatment**	**baseline**	**after pemafibrate treatment**	**baseline**	**after pemafibrate treatment**
Group2	87.7±15.6	85.5±13.3	6.7±1.1	6.6±0.7	304.5±186.5	157.2±110.2
	p=0.13		p=0.95		p<0.01	
	**baseline**	**after pemafibrate treatment**	**baseline**	**after pemafibrate treatment**	**baseline**	**after pemafibrate treatment**
Group3	73.4±10.8	73.0±9.7	6.4±0.5	7.1±0.8	259.6±97.89	250.8±82.2
	p=0.89		p=0.22		p=0.08	

A statistical improvement of clinical items were observed in BW, HbA1c, TG of group1 patients with primary treatment, and TG of group2 patients with pemofibrate treatment.

As resistance increases, it is clear that multiple drugs are needed.

### Treatment resistance-related clinical parameters

**Figure 3.**
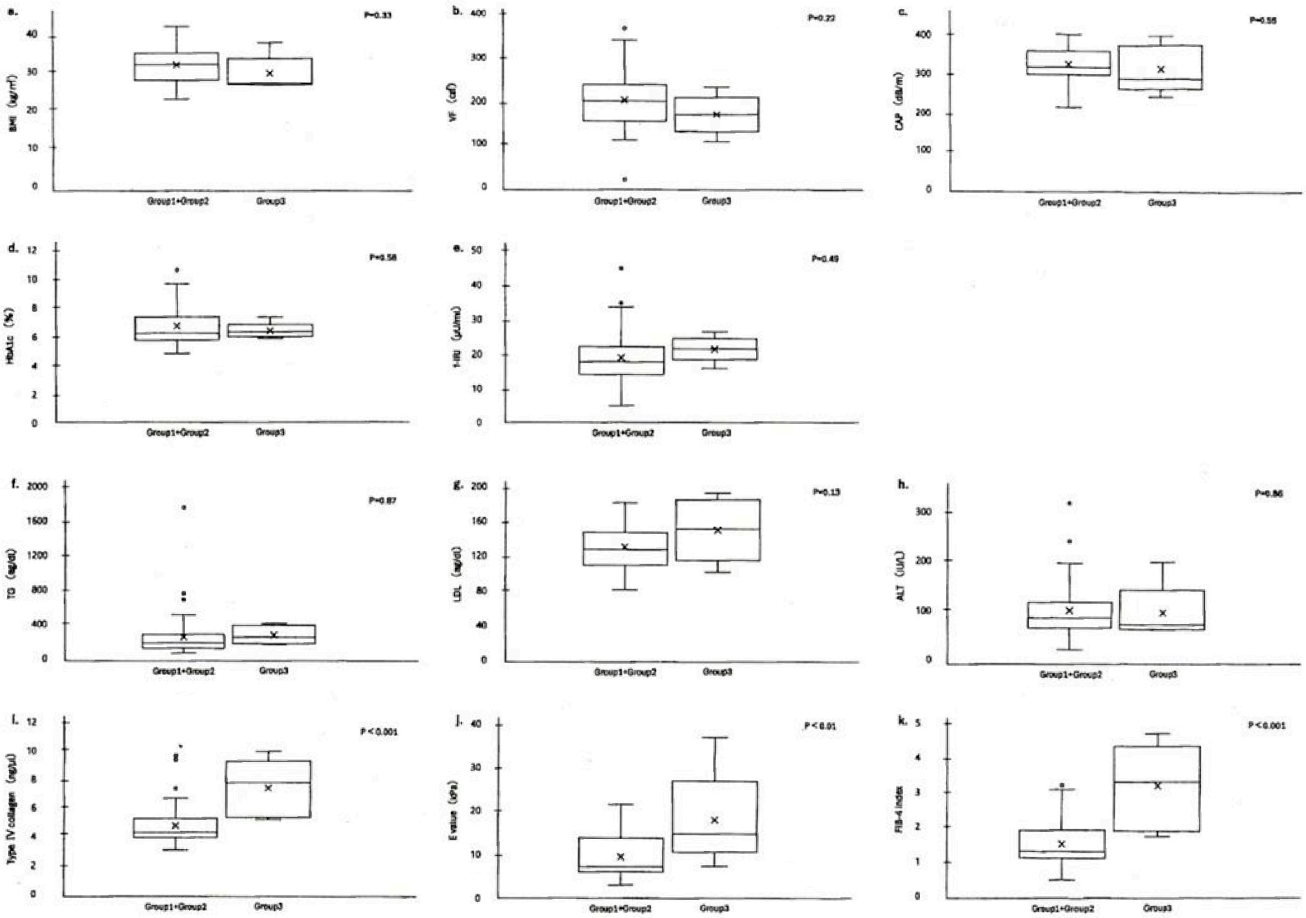
Identification of treatment resistance-related clinical parameters Boxplots (median, upper and lower quartiles, range and outliers [circles]), comparing parameter level distributions of treatment-sensitive group 1+group 2 and those of resistant group 3. a)~h) Baseline levels of characteristics (BMI, VF, CAP, HbA1c, f-IRI, TG, LDL cholesterol, and ALT) were demonstrated, comparing treatment-sensitive group 1+group 2 and resistant group 3. These characteristic levels had no difference. Note: P-values were calculated by use of t-test, but there were not significant differences of all. i)~k) Baseline levels of characteristics (type IV collagen, E value, and FIB-4 index) were demonstrated, comparing treatment-sensitive group 1+group 2 and resistant group 3. These characteristic levels had differences between two. Note: P-values were calculated by use of t-test, and each of them was p < 0.001 (type IV collagen), p < 0.01 (E value) and p < 0.001 (FIB-4 index). There were significant differences in P-value. ALT: alanine aminotransferase; BMI: body mass index; CAP: controlled attenuation parameter; E value: elasticity value; f-IRI: fasting immunoreactive insulin; TG: triglyceride; VF: visceral fat.

Based on the results of group 1+group 2 (treatment-effective) and group 3 (treatment-resistant), we identified clinical parameters to determine which one was responsible for treatment resistance. The characteristics (BMI, VF, CAP, HbA1c, f-IRI, TG, LDL cholesterol, and ALT) were demonstrated in Figure 3, comparing the level distribution of group 1+group 2 and that of group 3. The differences between treatment-effective group 1+group 2 and treatment-resistant group 3 were statistically tested, using the t-test. As a result, the differences for metabolic markers (BMI, VF, CAP), DM-related ones (HbA1c, f-IRI), lipid metabolism ones (TG, LDL cholesterol), and hepatitis one (ALT) were not significant; BMI (p = 0.33), VF (p = 0.22), CAP (n = 0.55), HbA1c (p = 0.58).

f-IRI (p = 0.49), TG (p = 0.87), LDL cholesterol (p = 0.13), and ALT (p = 0.86). None of them was statistically significant (p > 0.01; Table 4). The baselines of these parameters BMI, VF, CAP, HbA1c, f-IRI, TG, and ALT were not related to the treatment resistance. Therefore, the values of clinical parameters (baseline before treatment) were not helpful for a choice of treatment.

**Table 4.** Statistical Analysis of Treatment Resistance-Related Parameters (The Summary of the Results in Figure 3).

Clinical parameters		p values (t test/chi-square test＊)	Cutoff value
		Group1-Group2 vs Group3	
		(treatment effective) (treatment resistant)	
Obesity	BMI	0.33	
Metabolic syndrome	VF(visceral fat) (CT)	0.22	
	CAP (fibroscan)	0.55	
Diabetes Mellitus	HbA1c	0.58	
	f IRI	0.49	
Lipid metabolism	Triglyceride	0.87	
	LDL cholesterol	0.13	
Hepatitis	ALT	0.86	
Liver fibrosis/hepatitis	E value(fibroscan)	<0.01	13.7kPa
Liver fibrosis	type IV collagen	<0.001	5.3ng/ml
	stage (fibrosis)	0.01 (Stage1+Stage2/Stage3)^*^	Stage2
		0.031(Stage1/Stage2+Stage3)^*^	
	FIB4-index	<0.001	1.89

The differences between baseline levels of treatment sensitive group1+group2 and treatment resistant group3 were statistically examined, using the t test. The characteristic levels of BMI, VF, CAP, HbA1c, f IRI, TG, LDL cholesterol and ALT had no difference between two groups (sensitive and resistant). As a results, there were no significant differences in all. The baseline levels of type IV collagen, E value and FIB-4 index had differences between sensitive groups and resistant one. There were significant differences with use of t test. Moreover, there was significant difference with use of chi square test, stage2 (liver fibrosis related) was significant. BMI body mass index, CAP controlled attenuation parameter, f IRI fasting immunoreactive insulin, ALT alanine aminotransferase

In contrast, the differences between treatment-effective group 1+group 2 and treatment-resistant group 3 for 4 parameters (type IV collagen, E value, FIB-4 index, and MASH stage) were significant; type IV collagen (p < 0.001), E value (p < 0.01), FIB-4 index (p < 0.01), and MASH stage 2 (p < 0.01), shown in Figure 3 and Table 4. These 4 parameters were all related to fibrosis and MASH stage. These data suggested that those were to take responsibility for treatment resistance (Figure 3) and therefore, the treatments for MASH patients should be started in the early stage before fibrosis progression.

### ROC curves

**Figure 4.**
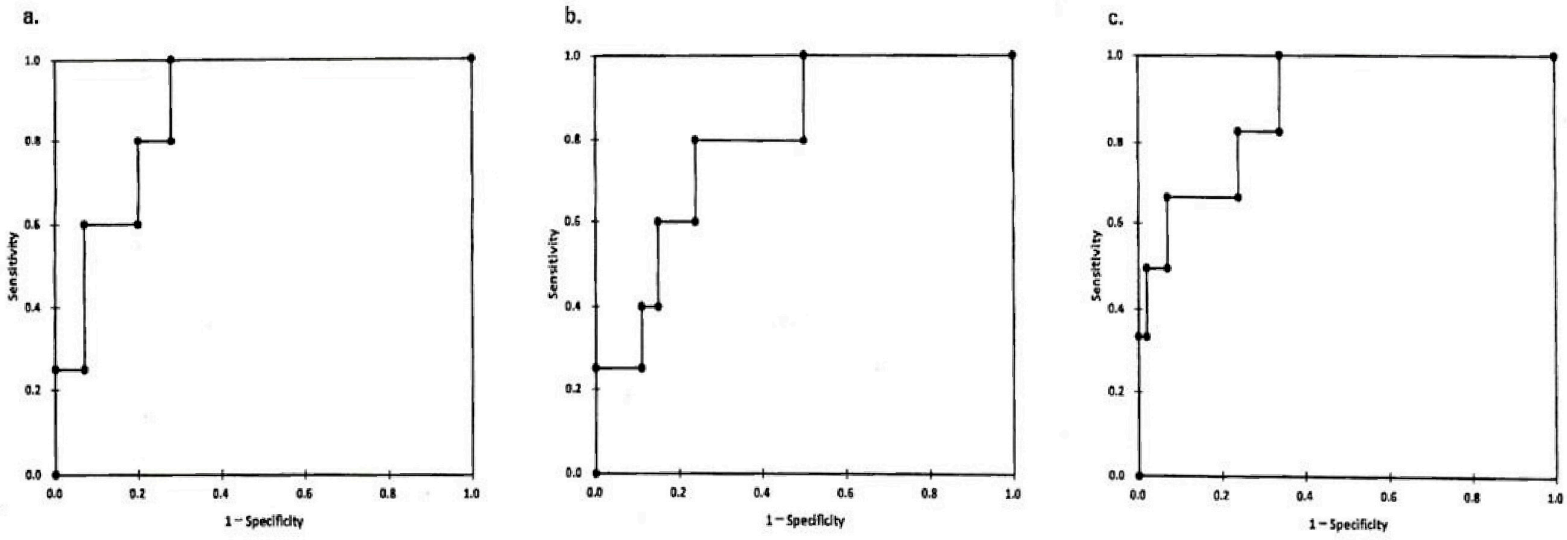
ROC analysis of (a) type IV collagen. The sensitivity and specificity of type IV collagen were determined in the study to discriminate type IV collagen of treatment-sensitive patients (group 1 + group 2) from that of treatment-resistant group 3. (b), (c) The sensitivity and specificity of E value (b) and FIB-4 index (c) were determined in the same study to discriminate. Cutoff values were determined 5.3 ng/ml for type IV collagen (a), 13.7 kPa for E value (b) and 1.89 for FIB-4 index (c), respectively. E value: elasticity value; FIB-4: age (year) x AST (IU/L)/platelet count (10^4^/L) x ALT (IU/L)^1/2^; ROC: receiver operating characteristic.

We constructed ROC curves by plotting. Type IV collagen is presented in Figure 4a. The cutoff value was 5.3 ng/ml between treatment-sensitive patient groups (group 1+group 2) and treatment-resistant group (group 3). In the same way, the cutoff values were determined at 13.77 kPa for the E value, 1.89 for the FIB-4 index, and stage 2 for MASH staging (Figure 4b and 4c). These cutoff values obtained in this study were equivalent to those in stage 2 fibrosis, shown in positive pattern recognition system ^(14)^ (type IV collagen 5.9 ± 1.0 ng/ml, E value 13.3 ± 6.2 kPa, in stage 2~3).

In summary, the treatment for MASH patients should be started from an early stage (around stage 2 before becoming stage 3, FIB-4 index before 1.89). The second important thing is that the baseline levels of ALT (inflammation marker), TG (lipid metabolism), DM marker, and BMI (obesity marker) were useless for a prediction of treatment options and of no use to determine whether there is treatment resistance or not.

## Discussion

Many treatments and medications ^(20)^ have been proposed for patients with MASLD/MASH. However, most of them were the results of the efficacy of a single medication and for a short duration of treatment. The present study revealed that the efficacy rate was over 90% for all MASH patients using multiple means, multidrug therapy, and long-term duration and to stop the progression of liver fibrosis by suppressing hepatic inflammation (destruction of hepatocytes).

ALT (hepatitis), E value (hepatitis/fibrosis) and type IV collagen (fibrosis) as markers for evaluation of treatment effect were selected ^(1), (4)^. We have reported that the use of these 3 markers makes it possible to decide the stage of MASH ^(14)^. When liver biopsies were performed twice on several patients before and after treatment in this study, treatment-sensitive patients showed no change or no improvement of the MASH stage, but resistant patients showed fibrosis stage up. From the above, the reduction or stabilization of 3 marker levels after treatment could be set as an improvement target as well as a goal to prevent it from worsening. In addition to BW control, vitamin E, SGLT2i (primary treatment), and then pemafibrate (second treatment) were used as medication. The reason why we used these medications is that there were many reports for MASH treatment about a single use and in short-term (1-2 years) follow-up study and we had used them to be convinced of their effectiveness in pilot studies.

Vitamin E, beyond its antioxidant effects, has been implicated in the regulation of the inflammatory response through several enzymes involved in signal transduction ^(5), (6), (21)^. SGLT2i treatment contributes to MASH by reducing hyperglycemia, improving systematic insulin resistance, elevation of caloric loss, and reduction of BW due to glycosuria. It plays a hepatoprotective effect through the reduction of hepatic de novo lipogenesis, hepatic inflammation, apoptosis, ER-oxidative stress, and increase of hepatic beta-oxidation ^(22), (23), (24)^.

Pioglitazone has a role in improving insulin resistance, glucose uptake into muscle and fat tissue, and suppressing gluconeogenesis in the liver ^(5)^.

As there were side effects (BW up, induction of heart failure) for clinical use, we rarely use it. Pemafibrate, as a medication of secondary treatment, activates peroxisome proliferator-activated receptor-α modulator (PPARα) that promotes lipid turnover and induces fatty acid β-oxidation, and suppresses TG synthesis in the liver ^(9)^. In contrast, it induces TG hydrolysis and decreases serum TG level ^(10), (25), (26)^. The reductions in ALT, E value, and type IV collagen levels were observed, comparing the levels (baseline, before treatment) and those after treatment in group 1 (n = 35) (Figure 2a). The differences of 3 marker levels were significant, according to Wilcoxon’s signed-rank test; ALT (p < 0.001), E value (p < 0.01) and type IV collagen (p < 0.01). Therefore, group 1 patient (n = 35) were sensitive to primary treatment. The proportion of sensitive patients is only 69%, while the levels of markers were reduced to normal levels.

From the above, the primary treatment (BW control, vitamin E, and SGLT2i) using many drugs for 5 years could not control hepatic inflammation and fibrosis of all MASH patients, and as a result, 31% of them became treatment-resistant. As an additional treatment, pemafibrate was examined for its effectiveness for 16 resistant patients. Significant reductions of ALT (p < 0.01) and type IV collagen levels (p = 0.045) were observed, using pemafibrate treatment (Figure 2c). However, there was no statistical difference in E value levels (baseline and those after treatment). In contrast, the differences in E value levels between pretreated and post-treated patients with pemafibrate showed significant (p = 0.05). It is conceivable that E values are extremely elevated in primary treatment.

This study made it clear that 90% of MASH cases could achieve recovery by improving multiple factors and pathways including BW, hepatic inflammations, insulin resistance, and TG metabolism.

Treatments for conventional liver diseases (direct antiviral agents (DAAs) for hepatitis C, nucleic acid analogue (NA) for hepatitis B, and stopping drinking for alcoholic steatohepatitis) used to be based on one-to-one correspondence; however, the MASH treatment plan is completely different and complicated. Moreover, the baseline levels (before treatment) of ALT (inflammation marker), HbA1c, f-IRI (DM marker), TG (lipid metabolism), and BMI (obesity marker) were useless for a prediction of treatment options and of no use to determine whether there is treatment resistance or not.

This study has revealed that type IV collagen, E value, FIB-4 index, and MASH stage may be responsible for treatment resistance. Furthermore, cutoff values were statistically determined by the ROC curves; type IV collagen 5.3 ng/ml, E value 13.7 kPa, FIB-4 index 1.89, and MASH stage 2 or less.

### Limitations

Our study has several limitations. One is that the number of MASH patients was limited. We are ready to organize clinical trials to be conducted by cooperative groups. Though limitations exist, we would like to propose the details of MASH treatment, especially multidrug therapy. This study has indicated the trends in the efficacy of multidrug therapy.

Another point of limitation is that the studies for an early-onset MASH (<40 years old) and an old-aged MASH (>60 years old) would be necessary since the efficacy of treatment was different from each other. We are ready to summarize this point. As described in the Results, the features of early-onset MASH patients were 10% diabetes prevalence, high rate of impaired glucose tolerance, high insulin resistance, and obese condition. Therefore, the best treatment was BW control for this group. In contrast, however, the features of old-aged MASH patients were long duration of illness (20-30 years), 50% of diabetes prevalence, insulin resistance down, BW tendency down, and liver fibrosis stage 3, stage 4 (liver cirrhosis), and HCC (hepatocellular carcinoma) patients included. Therefore, the treatment of this group was complicated and different. From the above, it was better to select middle-aged MASH patients for the typical treatment model to prevent it from getting worse.

In conclusion, the treatment for MASH patients should be started from an early stage (type IV collagen <5.3 ng/mL, E value <13.7 kPa, FIB-4 index <1.89, and MASH stage 2 or less).

## Article Information

### Conflicts of Interest

None

### Acknowledgement

This study was carried out based on the research collaboration contact between Tenshi Hospital and Sapporo Medical University. The authors thank the following individuals for assistance in the preparation of this manuscript: Aiko Hayashi, Hiroshi Makino, and Masanobu Huruichi.

### Author Contributions

MT: study concept, design, and analysis, drafting of the manuscript, TT: acquisition of data, critical revision of the manuscript for important content. HT, SN, MS, HI: critical revision of the manuscript for important content. SS: critical revision of the manuscript for important intellectual content, approval of the final version. NA: study concept and design, statistical analysis. HN: study supervision. All authors read and approved of the final manuscript.

### Approval by Institutional Review Board (IRB)

Approval code: 160-2022. Name of institution: Tenshi Hospital. Date of approval: August 19, 2022.

### Informed Consent

Although informed consent was not obtained from the participants, they were provided with an opportunity to deny participation by posting the opt-out document.
